# Ovulation Induction Changes Epigenetic Marks of Imprinting Genes in Mice Fetus Organs 

**DOI:** 10.22074/cellj.2021.6953

**Published:** 2021-03-01

**Authors:** Anahita Oveisi, Akbar Vahdati, Maryam Shahhoseini, Raha Favaedi, Saman Maroufizadeh, Bahar Movaghar

**Affiliations:** 1 Department of Biology, Fars Science and Research Branch, Islamic Azad University, Fars, Iran; 2 Department of Biology, Shiraz Branch, Islamic Azad University, Shiraz, Iran; 3 Department of Genetics, Reproductive Biomedicine Research Center, Royan Institute for Reproductive Biomedicine, ACECR, Tehran, Iran; 4 Department of Cell and Molecular Biology, School of Biology, College of Science, University of Tehran, Iran; 5 School of Nursing and Midwifery, Guilan University of Medical Sciences, Rasht, Iran; 6 Department of Embryology, Reproductive Biomedicine Research Center, Royan Institute for Reproductive Biomedicine, ACECR, Tehran, Iran

**Keywords:** Epigenetic, Fetus, Imprinted Gene, Histone Modification, Ovarian Stimulation

## Abstract

**Objective:**

Genomic imprinting is an epigenetic phenomenon that plays a critical role in normal development of embryo. Using exogenous hormones during assisted reproductive technology (ART) can change an organism hormonal profile and subsequently affect epigenetic events. Ovarian stimulation changes gene expression and epigenetic pattern of imprinted genes in the organs of mouse fetus.

**Materials and Methods:**

For this experimental study, expression of three imprinted genes H19, Igf2 (Insulin-like growth factor 2) and Cdkn1c (Cyclin-dependent kinase inhibitor 1C), which have important roles in development of placenta and embryo, and the epigenetic profile of their regulatory region in some tissues of 19-days-old female fetuses, from female mice subjected to ovarian stimulation, were evaluated by quantitative reverse-transcription PCR (qRT-PCR) and Chromatin immunoprecipitation (ChIP) methods.

**Results:**

H19 gene was significantly lower in heart (P<0.05), liver (P<0.05), lung (P<0.01), placenta (P<0.01) and ovary (P<0.01). It was significantly higher in kidney of ovarian stimulation group compared to control fetuses (P<0.05). Igf2 expression was significantly higher in brain (P<0.05) and kidney (P<0.05), while it was significantly lower in lung of experimental group fetuses in comparison with control fetuses (P<0.05). Cdkn1c expression was significantly higher in lung (P<0.05). It was significantly decreased in placenta of experimental group fetuses rather than the control fetuses (P<0.05). Histone modification data and DNA methylation data were in accordance to the gene expression profiles.

**Conclusion:**

Results showed altered gene expressions in line with changes in epigenetic pattern of their promoters in the ovarian stimulation group, compared to normal cycle.

## Introduction

During prenatal stages of development, specific parental gene or cluster of genes are widely expressed monoallelically and termed "imprinted genes" (1). Expression of these genes is down-regulated after birth (1). Although imprinted genes occupied a small subset of the genome, they play critical roles for normal development of organisms (1). *H19, Igf2* (Insulin-like growth factor 2) as well as *Cdkn1c* (Cyclin-dependent kinase inhibitor 1C) are the most frequently studied imprinted genes (2). *H19* gene produces a non-coding RNA as a trans-regulator of a group of co-expressed imprinted genes, to control fetal and early postnatal growth in mice (3). One of these co-expressed imprinted genes is *Igf2* gene, which plays major role in promoting embryonic/ placental growth and development (4). Like *Igf2, Cdkn1c* is expressed in trophoblast cells. It is a cell cycle inhibitor and a negative regulator of cell proliferation. It is clear that orchestrated regulation of the imprinted genes network promotes and guarantees normal embryo development (5).

Imprinted genes mainly are regulated by epigenetic mechanisms including DNA methylation, interfering RNAs (including miRNA, piRNA, siRNA) and histone modification to promote normal development of embryo. It has also been shown that some defects in genomic imprinting can cause infertility (6, 7).

The major epigenetic process that is recognized to be associated with imprinted genes in both gametes and developing embryos is DNA methylation (8). It is one of the most studied epigenetic mechanisms that can affect activity of DNA segment and gene expression without changing its sequence. There are three epigenetic mechanisms that control gene expression:

1. DNA methylation is a process in which the methyl group is added to specific dinucleotide CpG sites in the genome. Hypermethylation of these sites in the genome leads to gene suppression, while hypomethylation of them can cause gene over-expression. Sites of DNA methylation are engaged by various proteins, containing methyl-CpG binding domain (MBD) proteins which recruit enzymatic machinery to create silent chromatin (9). Among them, Methyl CpG binding protein 2 (MeCP2) as a DNA methylation "reader" protein specifically binds to methylated DNA regions and typically can be detected by chromatin immunoprecipitation techniques, as an epigenetic marker for DNA methylation (10). 2. interfering RNAs (including miRNA, piRNA, siRNA), which describes epigenetic and posttranscriptional regulation of transposons and genes (7). 3. histone modification which describes posttranslational modifications altering interaction of the histones with DNA and nuclear proteins (11). 

H3K9 (lysine 9 of histone 3) is an important position in the genome, as balance between its acetylation (H3K9ac) and deacetylation can regulate gene expression. H3K9ac and H3K9 trimethylation (H3K9me3) could have critical roles in epigenetic regulation of gene expression. Acetylation of this position causes opening of chromatin and mediating gene transcriptional activity. In contrast, its deacetylation (which is usually simultaneous with methylation) results in gene transcriptional repression. These two situations cause chromatin structure to be accessible or inaccessible for transcription. In addition, "bivalent marks" of H3K4me3 and H3K27me3(trimethylated lysine 4 and 27 on histone H3) are respectively activating and repressing histone marks that regulate gene expression level (12).

Histone codes like H3K9ac and H3K4me3 cause gene up-regulation and others such as H3K9me2 and H3K27me3 lead to gene repression (11). Some studies have shown a link between transcription of imprinted differentially methylated regions and removal insertion of histone modifications (13).

Patients undergo ovarian stimulation through *in vitro* fertilization (IVF) procedures, using high doses of exogenous gonadotropins, to enable retrieval of multiple oocytes in one cycle and this stimulation may affect oogenesis, oocyte/embryo quality and prenatal outcomes (14).

*In vitro* studies showed that ovarian stimulation disrupts and delays development of one- or two-cell mouse embryos into blastocysts (15, 16). *In vivo* studies are concordant, indicating that ovarian stimulation delays embryo development (16, 17). Study of Sato et al. in the human and mouse suggest that ovarian stimulation/ superovulation can lead to the production of oocytes without correct primary imprint. They demonstrated that the results of studies on human are inconsistent with mouse studies (18).

Ovarian stimulation is the most important cause of multiple pregnancies and consequently low birth weight, increased risk of miscarriage, growth retardation and preterm delivery (14). Finally, ovarian stimulation has been shown to be the cause for imprinting defects. For example, overexpression of gene *IGF2* with paternal imprinting in the placenta have been correlated with fetal growth restriction in humans (14). 

Manipulations in hormonal profile, reproductive system and gametes of organism during assisted reproductive technology (ART) can affect epigenetic events of genome, e.g. genomic imprinting (19). Assessment of the relationships between epigenetics, genomic imprinting and ART offers new perspectives in the understanding of molecular bases of infertility and ART failure. This study focuses on understanding expression changes of the important developmental imprinted genes (*H19, Igf2* and *Cdkn1c*) and the epigenetic situation of their regulatory region in a set of tissues from 19-days-old fetuses of mice subjected to ovarian stimulation.

## Materials and Methods

### Ovarian stimulation of naval medical research institute mice and obtaining embryos

In this experimental study, assessment was performed on two groups of 19-days-old fetuses of Naval Medical Research Institute (NMRI) mice (Pasteur Institute, Iran). In the first group, 16 fetuses were collected from uterus of four female mice, subjected to ovarian stimulation before gestation. The second group consisted of the 16 fetuses obtained from female mice with natural pregnancy, as control. Four fetuses were excluded and 12 fetuses were included for gene expression assessments in this study. Female mice were kept in the animal house of Royan Institute (Tehran, Iran) at temperature of 19-23˚C and humidity of 40-50%, 12 hours light (6 am- 6 pm) and 12 hours darkness. For growth and maturation of ovarian follicles in 8-weeks-old female mice of the first group, 7.5 IU PMSG (pregnant mare serum gonadotropin) hormone (Folligon; Invert, Belgium) followed 48 hours later by 7.5 IU of hCG (Human chorionic gonadotropin) hormone (Organon, Netherlands) were administered. Female mice of the both groups were mated with NMRI male mice (20). After formation of vaginal plaque (mating indication) females were isolated and sacrificed on the 19^th^ day of pregnancy. The fetuses were obtained, and seven different tissues of each fetus -including brain, lung, heart, liver, kidney, ovary and placenta- were collected. Few parts of tissues were preserved in RNA later (Ambion, USA) reagent at -70˚C for future RNA isolation and the rest of tissues were preserved at -70˚C for later epigenetic evaluations (20, 21). This study was approved by the Institutional Ethics Committee of Royan Institute (Tehran, Iran) on 2^nd^ July 2014 (code: EC/93/1038).

### RNA isolation and quantitative reverse-transcription PCR

RNA isolation and qRT-PCR quantitative reveres transcription PCR (qRT-PCR) were performed on tissues using the RNeasy micro kit (Qiagen, USA) according manufacture’s instruction. Quantification of mRNA levels of imprinted genes (*H19, Igf2* and *Cdkn1c*) was performed in duplicates by qRT-PCR on a StepOnePlus Real-Time PCR System (Applied Biosystems Instruments, USA) using SYBR Green master mix (Applied Biosystems). Designed primers are listed in Table 1. Condition of qRT-PCR amplification was 95˚C for 10 minutes, followed by 40 cycles of 95˚C for 15 seconds and 60˚C for 60 seconds. Gene expression data were analyzed using 2^-ΔΔCt^ quantitative method to estimate relative fold change values in comparison with *Gapdh* gene, as an endogenous control [mean ± SEM, (20)].

### Chromatin immunoprecipitation real time polymerase Chain Reaction analysis

Chromatin immunoprecipitated (ChIP) PCR experiments were performed, using a histone ChIP kit according to manufacturer’s instruction (Diagenode, Belgium). Briefly, all tissues were suspended in PBS. Then formaldehyde (1% final concentration) was added to the samples and then incubated gently on a shaking platform for 10 minutes at room temperature. In the next step, glycine was added into the samples to reach final concentration of 125 mM to quench the cross-linking reaction of formaldehyde. After washing the samples with PBS, lysis buffer was added and sonicated for 10 minutes (30 "on/30" off; Bioruptor sonication system, Diagenode) to get soluble sheared chromatin. After 5 minutes centrifugation at 14000 g, the supernatant was divided into six parts (Each part 10 µl). One part was used as input control, and the other 5 parts were incubated with 1µl of anti-H3K9ac, anti-H3K9me2, anti-H3K4me3, anti-H3K27me3 and anti-MeCP2 antibodies (1µg/µl; Abcam, UK) overnight at 4˚C on rotator. Immune complexes were washed three times using 100μl ice-cold washing buffer and then incubated on a rotating wheel for 4 minutes at 4˚C. Using a magnetic rack the beads were captured and immediately treated with 100μl DNA isolation buffer. The recovered DNA from immunoprecipitated fractions and total chromatin input, were quantified by real-time PCR. Data were expressed as fold enrichment of DNA associated with different immunoprecipitated histone modifications. DNA methylation was expressed as relative to a 1/100 dilution of input chromatin. Quantitative real-time PCR was carried out on a step one plus Real-Time PCR System (Applied Biosystems) using SYBR Green PCR master mix (Applied Biosystems) and designed primers (Table 1). The condition was 95˚C for 10 minutes; and 40 cycles of 95˚C for 15 seconds, 60˚C for 45 seconds. Results were normalized to input DNA and expressed as (%) input, which means percentage of enriched DNA associated with immunoprecipitated chromatin [mean ± SEM, (21)].

**Table 1:** Primers used in this study


Gene	Primer sequence (5´- 3´)	Product size (bp)	Location

Primers used in qRT-PCR			
*H19*	F: GCAGGAATGTTGAAGGAC	132	NR-001592
	R: CGGGATGAATGTCTGGCTC		
*Igf2*	F: AGTTCTGCTGCTGCTTATTG	168	NM-010514
	R: CTACCTGGCTAGTCATTGG		
*Cdkn1c*	F: TCCAGCGATACCTTCCCA	148	NM-009876
	R: GTCCACCTCCATCCACTG		
*Gapdh*	F: GACTTCAACAGCAACTCCCAC	125	NM-001289726
	R: TCCACCACCCTGTTGCTGTA		
Primers used in ChIP real time PCR			
*H19*	F: AAGGGAACGGATGCTACC	85	Promoter
	R: CTGGGATATTGCTGGGAATG		
*Igf2*	F: GTCACCACTGTATCATTCTGC	152	DMR1
	R: TGCTAACACACGCCTATCC		
*Cdkn1c*	F: GTTCGCTTGCTCTCAGTC	201	Promoter
	R: CATTATGCTAATCGTGAGGAGG		


### Statistical analysis

Data analysis was carried out using IBM SPSS Statistics for Windows, Version 22.0 (IBM Corp., USA). In this study, continuous variables were expressed as mean ± SEM (standard error of mean). An independent t test was used to compare control and ovarian stimulation groups. All statistical tests were two-tailed and a P<0.05 was considered statistically significant.

## Results

### Alterations of gene expression in ovarian stimulation group

**Fig.1:**
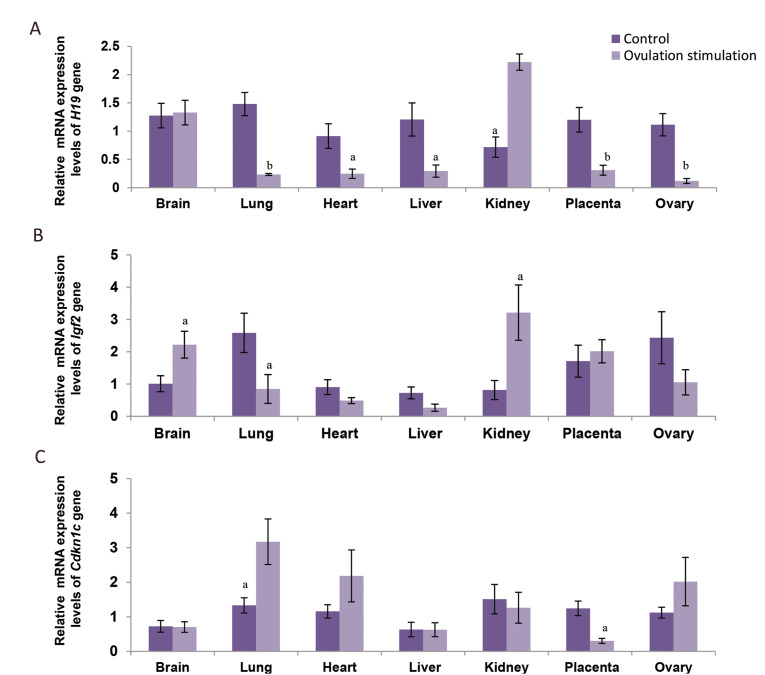
Relative mRNA expression of **A.** *H19*, **B. ***Igf2*, and **C.** *Cdkn1c* genes in brain, lung, heart, liver, kidney, placenta and ovary of fetuses from ovarian stimulation group (12 fetuses) in comparison with normal cycle fetuses (12 fetuses). Values are expressed as means ± SEM. The letters above the columns show significant difference between control and experimental groups. (a; P<0.05, b; P<0.01).

Relative mRNA expression levels of *H19* gene from all tissues except brain showed alteration in the ovarian stimulation group, compared to the control. This gene expression was decreased in lung (1.48 ± 0.41, 0.23 ± 0.16; P<0.01), heart (0.91 ± 0.23, 0.25 ± 0.09; P<0.05), liver (1.21 ± 0.3, 0.29 ± 0.12; P<0.05), placenta (1.2 ± 0.21, 0.31 ± 0.13; P<0.01) and ovary (1.11 ± 0.22, 0.12 ± 0.06; P<0.01) in the 19-days-old fetuses of the ovarian stimulation group compared to the control fetuses, respectively. However, kidney (0.72 ± 0.23, 2.22 ± 1.19) showed increased levels of *H19* in the experimental group, compared to control (P<0.05; Fig.1).

*Igf2* gene showed significantly higher levels of expression in brain (1.01 ± 0.25, 2.22 ± 0.42; P<0.05) and kidney (0.81 ± 0.29, 3.21 ± 0.86; P<0.05). While, it was significantly lower expressed in lung (2.59 ± 0.61, 0.91 ± 0.51) of ovarian stimulation group, than control fetuses (P<0.05; Fig.1). 

*Cdkn1c* showed significant increase in lung (1.33 ± 0.22, 3.17 ± 0.66; P<0.05) and significant decrease in placenta (1.24 ± 0.21, 0.3 ± 0.07; P<0.05; Fig.1). 

### Histone modification profile of the studied genes in the experimental group embryos

**Fig.2:**
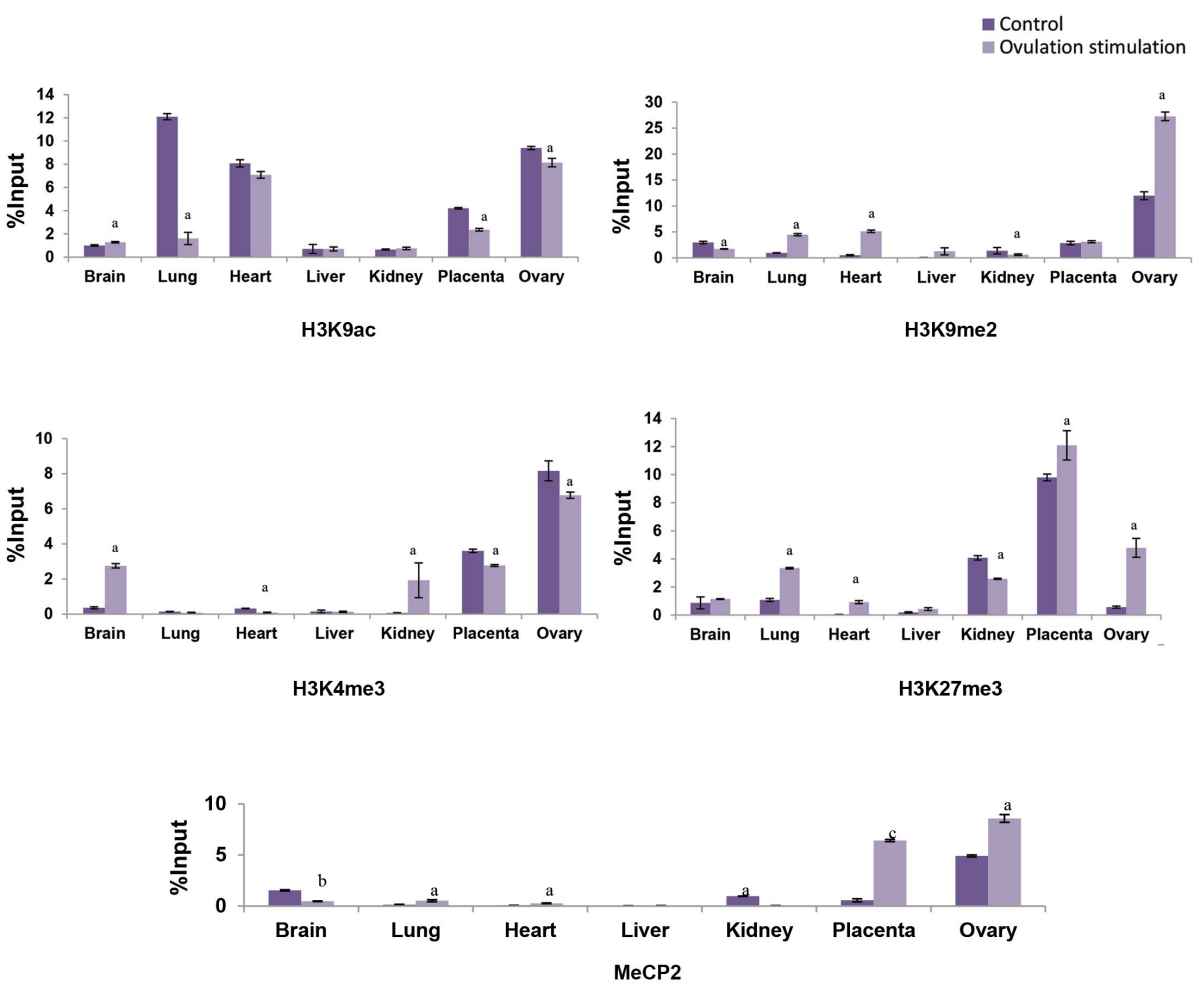
Incorporation of H3K9ac/me2 and H3K4/27me3 histone modifications and MeCP2 in regulatory region of *H19* gene in brain, lung, heart, liver, kidney, placenta and ovary tissues of ovarian stimulation fetuses (12 fetuses) versus control group (12 fetuses). Values are expressed as means ± SEM. The letters above the columns show significant difference between control and experimental groups. (a; P<0.05, b; P<0.01, c; P<0.001).

Our histone modification analyses, based on the ChIP data, were in accordance with the gene expression profile. Thus, higher incorporation of H3K9me2 gene repressing mark was detected in the *H19* promoter region of lung (P<0.05), heart (P<0.05) and ovary (P<0.05), while lower incorporation of it was detected in brain (P<0.05) and kidney (P<0.05) of experimental group fetuses compared to the control fetuses. Higher incorporation of H3K27me3 gene repressing mark was detected in promoter region of *H19* in lung (P<0.05), heart (P<0.05), placenta (P<0.05) and ovary (P<0.05), whilst lower incorporation of it was determined in kidney (P<0.05) of ovarian stimulation group compared to control. H3K9ac gene activating histone mark was significantly higher in promoter region of *H19* in brain (P<0.05), but it was significantly lower in lung (P<0.05), placenta (P<0.05) and ovary (P<0.05) of experimental group fetuses, rather than controls. H3K4me3 gene activating histone mark was significantly higher in promoter region of *H19* in brain (P<0.05) and kidney (P<0.05) but its expression was lower in heart (P<0.05), placenta (P<0.05) and ovary (P<0.05) of experimental group fetuses in comparison with control fetuses (Fig .2). CHIP analyses for DNA methylation showed higher incorporation of gene repressing mark of MeCP2, detecting in the promoter region of *H19* in lung (P<0.05), heart (P<0.05), placenta (P<0.001) and ovary (P<0.05). At the same time, lower incorporation in brain (P<0.01) and kidney (P<0.05) of experimental group fetuses was observed, in comparison with control fetuses (Fig .2). 

**Fig.3:**
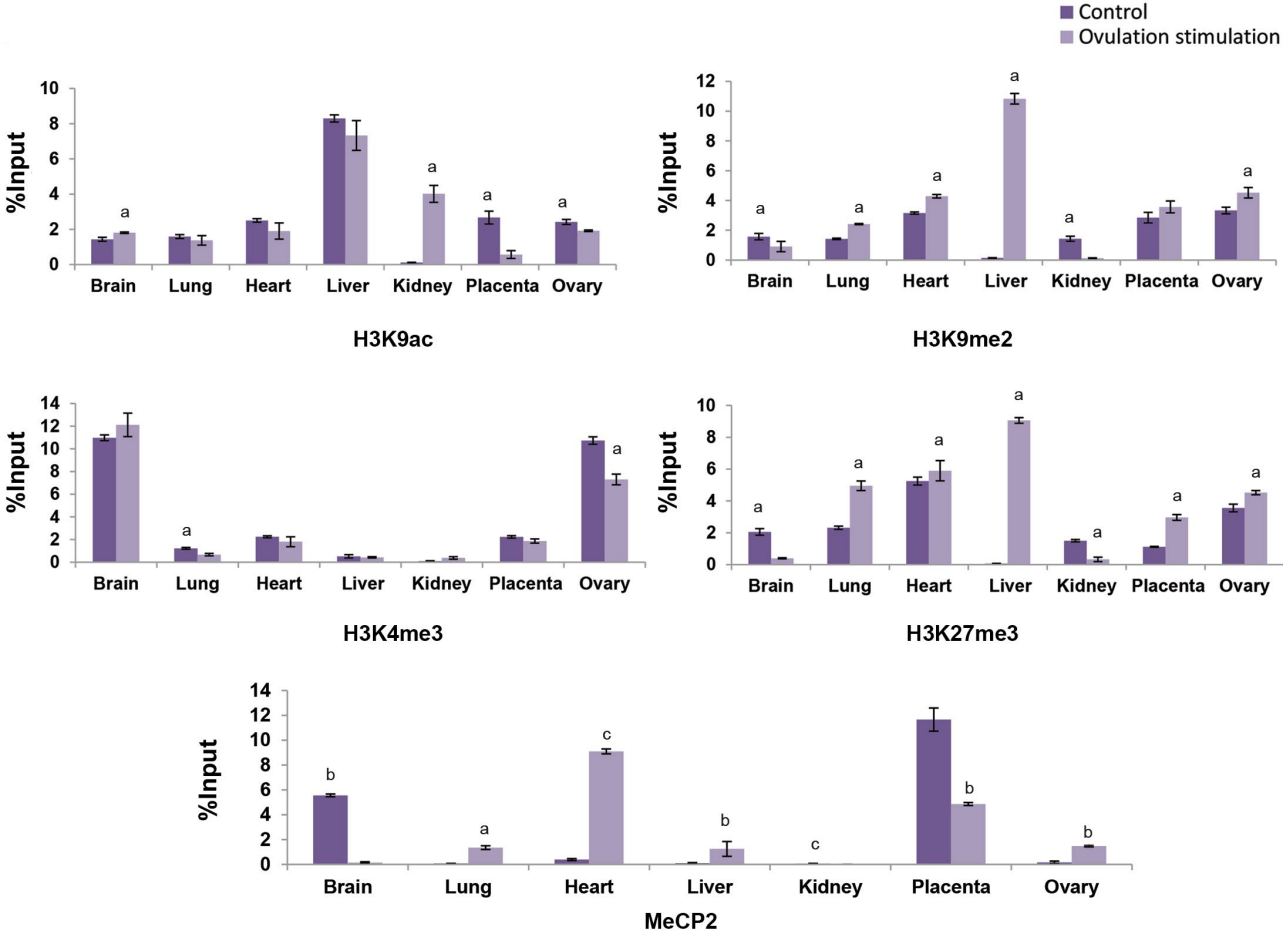
Incorporation of H3K9ac/me2 and H3K4/27me3 histone modifications and MeCP2 in regulatory region of *Igf2* gene in brain, lung, heart, liver, kidney, placenta and ovary tissues of ovarian stimulation fetuses (12 fetuses) versus control group (12 fetuses). Values are expressed as means ± SEM. The letters above the columns show significant difference between control and experimental groups. (a; P<0.05, b; P<0.01, c; P<0.001).

Higher incorporation of H3K9me2 gene repressing mark was detected in *Igf2* promoter region of lung (P<0.05), heart (P<0.05), liver (P<0.05) and ovary (P<0.05), while lower incorporation was detected in brain (P<0.05) and kidney (P<0.05) of ovarian stimulation group, compared to control. Higher incorporation of H3K27me3 gene repressing mark was detected in promoter region of *Igf2* in lung (P<0.05), heart (P<0.05), liver (P<0.05), placenta (P<0.05) and ovary (P<0.05), but lower incorporation of it in brain (P<0.05) and kidney (P<0.05) of ovarian stimulation group was detected, in comparison with control. H3K9ac gene activating histone mark was significantly higher in the *Igf2* promoter region of brain (P<0.05) and kidney (P<0.05) tissues, but it was significantly lower in placenta (P<0.05) and ovary (P<0.05) of the experimental group fetuses compared to the control fetuses. H3K4me3 gene activating histone mark was significantly lower in the *Igf2* promoter region of lung (P<0.05) and ovary (P<0.05) of the experimental group fetuses than control fetuses. Using ChIP experiment, DNA methylation studies showed higher incorporation of gene repressing mark of MeCP2 in the *Igf2* promoter region of lung (P<0.05), heart (P<0.001), liver (P<0.01) and ovary (P<0.01), while it was lower incorporated in brain (P<0.01), kidney (P<0.001) and placenta (P<0.01) experimental group fetuses, compared to control fetuses (Fig .3).

**Fig.4:**
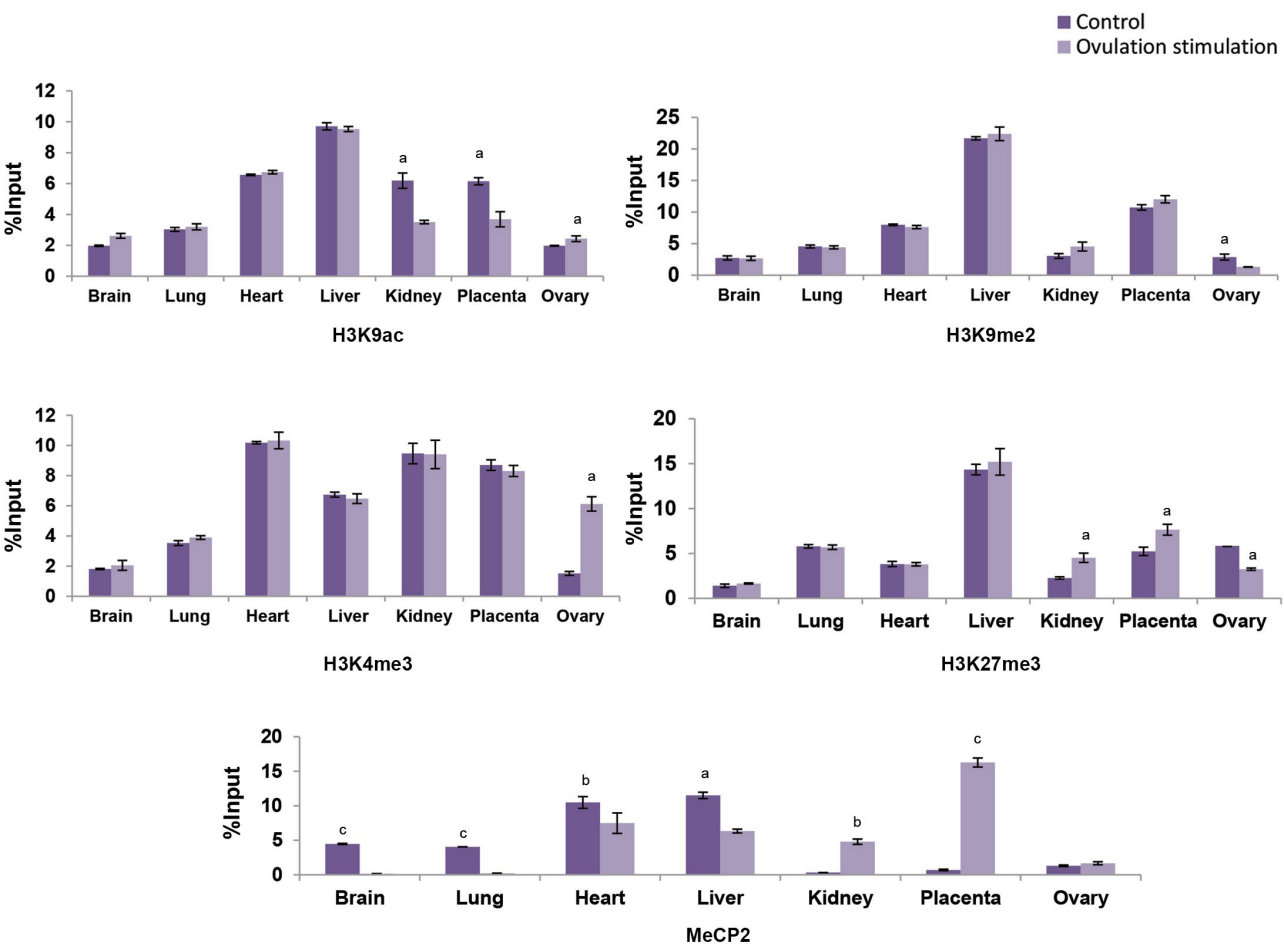
Incorporation of H3K9ac/me2 and H3K4/27me3 histone modifications and MeCP2 in regulatory region of *Cdkn1c* gene in brain, lung, heart, liver, kidney, placenta and ovary tissues of ovarian stimulation fetuses (12 fetuses) versus control group (12 fetuses). Values are expressed as means ± SEM. The letters above the columns show significant difference between control and experimental groups. (a; P<0.05, b; P<0.01, c; P<0.001).

H3K9me2 gene repressing histone mark was expressed significantly lower in promoter region of *Cdkn1c* in ovary (P<0.05) experimental group fetuses versus the control fetuses. Higher incorporation of H3K27me3 gene repressing mark was detected in promoter region of *Cdkn1c* in kidney (P<0.05) and placenta (P<0.05), but it was significantly lower in ovary (P<0.05) of the experimental group fetuses than the control fetuses. Higher incorporation of H3K9ac gene activating histone mark was detected in the promoter region of *Cdkn1c* in ovary (p<0.05), but it was significantly lower in kidney (P<0.05) and placenta (P<0.05) of the experimental group fetuses versus the control fetuses. H3K4me3 gene activating histone mark was significantly higher in the promoter region of *Cdkn1c* in the ovary (P<0.05) of experimental group fetuses compared to the control fetuses. Analysis of DNA methylation, using ChIP assay showed higher incorporation of gene repressing mark of MeCP2 in the *Cdkn1c* promoter region of kidney (P<0.01) and placenta (P<0.001). However, lower incorporation was detected in brain (P<0.001), lung (P<0.001), heart (P<0.01) and liver (P<0.05) of experimental group fetuses rather than control fetuses (Fig .4).

There was no significant difference between the fetus weight of ovarian stimulated and control groups, in our study (Table 2).

**Table 2:** Fetal birth weight in ovarian stimulated and natural cycle mice


Ovulation stimulation group fetuses		Control group fetuses	
Number of fetuses	Average weight of each fetus (g)	Number of fetuses	Average weight of each fetus (g)

79	1.53 ± 0.1	69	1.66 ± 0.07


## Discussion

Exogenous gonadotropins, used in ART cycles, could have negative effect on gene expression and consequently embryo development and growth (14). Both of the animal and limited human studies showed high possibility of ovarian stimulation responsibility for modifications in maternal-affected gene products that are later required for imprinting maintenance in developing embryos (18).

Although most ART children do not show any abnormality, some studies have suggested the correlation between ART and increased incidences of low birth weight and also rare imprinting syndromes, such as BeckwithWiedemann syndrome (BWS), Angelman syndrome (AS) and etc. (22, 23).

*H19* gene produces a regulatory non-coding microRNA that plays a critical role in regulation of imprinted genes network. Previous studies have shown that this gene contributes to growth regulation of fetus and placenta, which controls expression of *Igf2* gene (24). Further, *H19* plays an important role in the development of the pre- and post-natal mice (25, 26). In our study, expressions of *H19* gene in the lung, heart, liver, placenta and ovaries of the experimental fetuses were reduced. Le et al. showed some long lasting disturbances in *H19/Igf2* expression and consequently developmental disturbances of skeletal muscle and liver in mice conceived by IVF (25). So growth defect and weight loss may be a result of *H19* down-regulation. Mono-allelic expression of *H19* in placenta of mice was seen in Fortier et al. study. They demonstrated that may be susceptible to perturbation after ovarian stimulation (27). Reversal *H19* imprinting in human and mouse oocytes upon ovarian stimulation was reported by Sato et al. (18).

Our histone modification analyses based on the ChIP data was in accordance to the gene expression profile; in the way that higher incorporation of gene repressing marks of MeCP2, H3K9me2 and H3K27me3 were detected in promoter region of *H19* in lung, heart and ovary of ovarian stimulation group compared to the controls. In placenta, only MeCP2 and H3K27me3 were increased, but both of the activating marks were decreased. An increasing in the both of activating histone marks in the *H19* regulatory region of brain from the experimental fetuses compared to the control fetuses was seen, but H3K9me repressing mark was decreased. In kidney H3K4me was increased and all repressing marks of this study were decreased in accordance to the up-regulation of this gene.

It is important to note that tissues derived from trophoblast, unlike ICM (inner cell mass) derived tissues, have no control mechanisms through gene expression and they are more susceptible to imprinting disorders. There are two hypotheses. In the first, environment affects more on extra-embryonic cells and this causes loss of imprinting in mid-gestation placentas. In the second, loss of imprinting may also occur in cells destined to form the embryo. Biallelic expression was occasionally observed in the embryo, suggesting mechanisms that safeguard imprinting might be more robust in the embryo, than the placenta. Probably a de novo lineage-restricted wave of methylation occurs in ICM, but not in trophectoderm lineages (28). This is consistent with the results of our study which showed extreme changes in gene expression of placenta.

Expression of *Igf2* was higher in brain and kidney of experimental group, compared to control fetuses. It is in accordance to higher levels of H3K9ac and lower levels of H3K9me2, H3K27me3 and MeCP2 in promoter of this gene from the experimental group fetuses compared to the control fetuses. Decreased gene activating mark (H3K4me3), in accordance with significant increase of repressor marks (H3K9me2, H3K27me3 and MeCP2) in promoter of *Igf2* from the experimental group versus control, can be a reason for down-regulation of this gene in lung of the former group. It is expected that such expression pattern of *Igf2* gene results in developmental changes in brain, kidney and lung postpartum. In a study by Ye et al., expression of *Igf2* in different organs of adult mice was investigated (29). Their study showed that expression in brain and heart is much higher than kidney and liver. However in our study the expression of *Igf2* in brain, kidney, heart and liver was apparently similar in natural cycle fetuses, but higher expression level was seen in lung and ovary. It can be due to the examination of the mouse fetus instead of adult mice. 

IGF1, IGF2 and their receptors are expressed in the fetal lung of humans, rodents and other species (30). There are increasing evidences suggesting that the IGF system plays a pivotal role in the development and differentiation of the fetal lung (31). Our study showed decrease in *Igf2* levels after ovarian stimulation in lung. Data were confirmed by histone modification results. Silva et al. showed that deficiency in *Igf2* expression in mice fetuses leads to delayed growth of lung (32). A study of Källén et al. (23) showed higher risk of respiratory problems in IVF-conceived babies (8.5% in IVF babies, compared to 2.99% among all infants born with other ART techniques like Intracytoplasmic sperm injection (ICSI), frozen embryos plus IVF babies) (23). So, expression of *Igf2* in IVF babies with respiratory problems may be disturbed. ICSI born babies had less respiratory problem than IVF born babies. This could be due to the male subfertility in ICSI cases and differences in the treated women of these groups. As it was shown in our study expression of *Igf2* is higher in experimental group kidneys, confirmed by MeCP2 decrease in its regulatory region and DNA methylation. *Igf2* is precisely regulated to ensure monoallelic expression in the most of tissues (33), emphasizing the importance of gene dosage. Normal development requires accurate expression and many disorders can be attributed to an abnormally high dose of *Igf2* caused by loss of imprinting (34). 

Occurrence of *Igf2* overexpression as a result of ovarian stimulation could be a reason of why IVF born children suffer from urogenital dysfunction and they need urogenital operations more than natural born children (35).

Some observations have shown that methylation changes can be a highly consistent feature of carcinogenesis and methylation errors are perhaps common observations in cancer (36). Wilms’ tumor, a childhood cancer of the kidney, is often associated with defects in the *WT1* gene, which encodes a transcriptional repressor of *Igf2* (37). Wilms’ tumor is also associated with mutations in the 11p15.5 region that affect *Igf2* imprinting: altered *Igf2* expression accounts for nearly 50% of all cases of Wilms’ tumor, and *Igf2* loss of imprinting is found in the vast majority (90%) of pathological cases (38).

Low expression of *Cdkn1c* in placenta was confirmed by high levels of MeCP2 and H3K27me3, in addition to low level of H3K9ac in promoter of this gene in ovarian stimulation group versus control. The expression of *Cdkn1c* in the lung of experimental group fetuses was higher than control group. Presence of histone modifications in promoter of *Cdkn1c* gene in lung showed no significant difference between these two groups, however, level of MeCP2 in promoter region of *Cdkn1c* was significantly decreased in the experimental group in comparison with control group. Equal expression of *Cdkn1c* in brain and liver of these two groups was in line with equal level of histone modifications in promoter of this gene. However, MeCP2 showed low level in promoter of *Cdkn1c* from the experimental group in comparison with the control group. 

*Cdkn1c* gene which is involved in development of embryo, encodes a protein that is an inhibitor of cyclicdependent kinase, cell proliferation and growth. It seems that *Cdkn1c* is a suppressor gene, while disturbance and alteration in its expression in human causes BeckwithWiedemann syndrome (39). Previous studies showed that expression level of *Cdkn1c* gene is related to developments of lung and kidney in mouse and human (40). In our study increased expression of *Cdkn1c*, as a growth inhibitor, in lung and its coordination with low expression of *Igf2* (which is involved in lung development) may leads to the limited growth of lung in the experimental group versus the controls. Our findings showed that expression of *H19, Igf2* and *Cdkn1c* were changed in lung and kidney following the ovarian stimulation and these changes are related to epigenetic alteration. Our findings indicated that protective mechanisms of ICM may act poorly in the lung and kidney. Additionally, specific mechanisms of transcriptional regulation in each tissue are under influence of the various environmental factors (35). The findings of this study indicated that ovarian stimulation strongly affects these mechanisms in these two organs.

## Conclusion

To summarize, the current study showed the impact of ovarian stimulation on the expression of genes and the epigenetic alterations- even at the end of gestation. Occurrence of these long lasting epigenetic changes may be a reason of growth and development disturbances, in future. Although many researchers believe that the fetus is able to eliminate and correct many of the problems created during its development, the present study showed that some of the problems could remain with fetus until birth and they can affect growth of the fetus.
